# A phase II trial evaluating the efficacy and safety of perioperative pirfenidone for prevention of acute exacerbation of idiopathic pulmonary fibrosis in lung cancer patients undergoing pulmonary resection: West Japan Oncology Group 6711 L (PEOPLE Study)

**DOI:** 10.1186/s12931-016-0398-4

**Published:** 2016-07-22

**Authors:** Takekazu Iwata, Ichiro Yoshino, Shigetoshi Yoshida, Norihiko Ikeda, Masahiro Tsuboi, Yuji Asato, Nobuyuki Katakami, Kazuhiro Sakamoto, Yoshinori Yamashita, Jiro Okami, Tetsuya Mitsudomi, Motohiro Yamashita, Hiroshi Yokouchi, Kenichi Okubo, Morihito Okada, Mitsuhiro Takenoyama, Masayuki Chida, Keisuke Tomii, Motoki Matsuura, Arata Azuma, Tae Iwasawa, Kazuyoshi Kuwano, Shuji Sakai, Kenzo Hiroshima, Junya Fukuoka, Kenichi Yoshimura, Hirohito Tada, Kazuhiko Nakagawa, Yoichi Nakanishi, West Japan Oncology Group

**Affiliations:** Department of General Thoracic Surgery, Chiba University Graduate School of Medicine, 1-8-1 Inohana, Chuo-ku, Chiba, Chiba Japan; First Department of Surgery, Tokyo Medical University Hospital, 6-7-1, Nishishinjuku, Shinjuku-ku, Tokyo, Japan; Department of Thoracic Surgery, National Cancer Center Hospital East, 6-5-1, Kashiwanoha, Kashiwa, Chiba Japan; Department of Thoracic Surgery, Ibaraki Prefectural Central Hospital, 6528, Kobuchi, Kasama, Ibaraki Japan; Division of Integrated Oncology, Institute of Biomedical Research and Innovation, 2-2, Minatojima minamicho, Chuo-ku, Kobe, Hyogo Japan; Department of Respiratory Surgery, National Hospital Organization Yokohama Medical Center, 3-60-2 Harajuku, Totsuka-ku, Yokohama, Kanagawa Japan; Department of Respiratory Surgery, National Hospital Organization, Kure Medical Center and Chugoku Cancer Center, 3-1, Aoyamacho, Kure, Hiroshima Japan; Department of General Thoracic Surgery, Osaka Medical Center for Cancer and Cardiovascular Diseases, 1-3-3 Nakamichi Higashinari, Osaka, Osaka Japan; Division of Thoracic Surgery, Department of Surgery, Kinki University Faculty of Medicine, 377-2, Ohnohigashi, Sayama, Osaka Japan; Department of Thoracic Surgery, Shikoku Cancer Center, 160, Minamiumemotomachiko, Matsuyama, Ehime Japan; Department of Pulmonary Medicine, Fukushima Medical University, 1, Hikarigaoka, Fukushima, Fukushima Japan; Department of Thoracic Surgery, Tokyo Medical and Dental University, 1-5-45 Yushima, Bunkyo-ku, Tokyo, Japan; Department of Surgical Oncology, Hiroshima University, 1-2-3, Kasumi, Minami-ku, Hiroshima, Hiroshima Japan; Department of Thoracic Oncology, National Kyushu Cancer Center, 3-1-1, Notame, Fukuoka Minami-ku, Fukuoka Japan; Department of General Thoracic Surgery, Dokkyo Medical University, 880, Kitakobayashi, Shimotsugagun Mibumachi, Tochigi Japan; Department of Respiratory Medicine, Kobe City Medical Center General Hospital, 2-1-1, Minatojimaminamimachi, Chuo-ku, Kobe, Hyogo Japan; Department of Thoracic Surgery, Hiroshima City Hospital, 7-33, Motomachi, Naka-ku, Hiroshima, Hiroshima Japan; Department of Pulmonary Medicine and Oncology, Graduate School of Medicine, Nippon Medical School, 1-1-5, Sendagi, Bunkyo-ku, Tokyo, Japan; Department of Radiology, Kanagawa Cardiovascular and Respiratory Center, 6-16-1, Tomiokahigashi, Kanazawa-ku, Yokohama, Kanagawa Japan; Department of Internal Medicine, Division of Respiratory Medicine, Jikei University School of Medicine, 3-25-8, Nishi-Shinbashi, Minato-ku, Tokyo, Japan; Department of Diagnostic Imaging and Nuclear Medicine, Tokyo Women’s Medical University, 8-1, Kawadacho, Shinjyuku-ku, Tokyo, Japan; Department of Pathology, Tokyo Women’s Medical University Yachiyo Medical Center, 477-96, Ohwada-Shinden, Yachiyo, Chiba Japan; Department of Pathology, Nagasaki University Graduate School of Biomedical Sciences, 1-12-4, Sakamoto, Nagasaki, Nagasaki Japan; Innovative Clinical Research Center, Kanazawa University Hospital, 13-1, Takaramachi, Kanazawa, Ishikawa Japan; Department of Thoracic Surgery, Suita Tokushukai Hospital, 21-1, Senriokanishi, Osaka, Japan; Department of Medical Oncology, Kinki University School of Medicine, 377-2, Ohnohigashi, Sayama, Osaka Japan; Research Institute for Diseases of the Chest, Graduate School of Medical Sciences, Kyushu University, 3-1-1, Maidashi, Higashi-ku, Fukuoka, Japan

**Keywords:** Idiopathic pulmonary fibrosis, Acute exacerbation, Lung cancer, Surgery, Pirfenidone

## Abstract

**Background:**

Idiopathic pulmonary fibrosis (IPF) often accompanies lung cancer, and life-threatening acute exacerbation (AE) of IPF (AE-IPF) is reported to occur in 20 % of IPF patients who undergo lung cancer surgery. Pirfenidone is an anti-fibrotic agent known to reduce disease progression in IPF patients. A phase II study was conducted to evaluate whether perioperative pirfenidone treatment could reduce the incidence of postoperative AE-IPF patients with lung cancer.

**Methods:**

Pirfenidone was orally administered to IPF patients who were candidates for lung cancer surgery; pirfenidone was dosed at 600 mg/day for the first 2 weeks, followed by 1200 mg/day. Surgery was performed after at least 2 weeks of 1200-mg/day administration. The primary endpoint was non–AE-IPF rate during postoperative days 0–30, compared to the null value of 80 %, and the secondary endpoint was safety. Radiologic and pathologic diagnoses of IPF and AE-IPF were confirmed by an independent review committee.

**Results:**

From June 2012 to January 2014, 43 cases were enrolled, and 39 were eligible (full analysis set [FAS]). Both pirfenidone treatment and surgery were performed in 36 patients (per protocol set [PPS]). AE-IPF did not occur in 37/39 patients (94.9 % [95 % confidential interval: 82.7–99.4 %, *p* = 0.01]) in the FAS, and in 38/39 patients (97.2 % [95 % confidential interval: 85.5–99.9 %, *p* = 0.004] in the PPS. A grade 5 adverse event (death) occurred in 1 patient, after AE-IPF; no other grade 3–5 adverse events were observed.

**Conclusions:**

Perioperative pirfenidone treatment is safe, and is promising for reducing AE-IPF after lung cancer surgery in IPF patients.

**Trial registration:**

This clinical trial was registered with the University Hospital Medical Information Network (UMIN) on April 16th, 2012 (Registration Number: UMIN000007774).

## Background

Idiopathic pulmonary fibrosis (IPF) is the most common interstitial lung disease, and has a histological appearance of usual interstitial pneumonia (UIP). IPF is known to be a risk factor for lung cancer [1] and is often observed in lung cancer patients. Life-threatening acute exacerbation (AE) of IPF (AE-IPF) may occur in association with cancer treatment, including radiotherapy, chemotherapy, and surgery, thereby severely restricting the therapeutic options for IPF-associated lung cancer. Among IPF patients who undergo surgery for lung cancer, postoperative AE-IPF is reported to occur in approximately 20 %, with an associated mortality of about 50 % [2–5]. According to nationwide research conducted by the Japanese Association of Thoracic Surgery, AE-IPF is the leading cause of death within 30 days after lung cancer surgery in Japan [6]. Thus far, no reports of successful preventative methods for postoperative AE-IPF have been published. Several treatments, including intraoperative fluid balance control [7], postoperative ulinastatin [8], and preoperative methylpredonisolone and sivelestat [9], have been reported to have the potential to prevent postoperative AE; however, these were all evaluated in small, single-institute prospective or retrospective studies. A Japanese multi-institutional retrospective large cohort study revealed that none of the potential prophylactic treatments tested, including steroids, sivelestat, and ulinastatin, demonstrated an ability to prevent AE [10].

The Assessment of Pirfenidone to Confirm Efficacy and Safety in Idiopathic Pulmonary Fibrosis (ASCEND) study group recently reported that pirfenidone, oral anti-fibrotic agent, significantly reduced disease progression, as reflected by lung function, exercise tolerance, and progression-free survival, in a phase III trial in patients with IPF [11, 12]. However, with respect to postoperative AE-IPF, the utility of pirfenidone has not yet been evaluated. Therefore, we conducted the phase II West Japan Oncology Group (WJOG) 6711 L study, called the “Perioperative pirfenidone for lung cancer with fibrosis evaluation: PEOPLE study,” to evaluate the safety and efficacy of perioperative pirfenidone treatment in the reduction of postoperative AE-IPF in patients with lung cancer.

## Methods

### Eligibility criteria

Patients were required to have the UIP/possible UIP pattern criteria published by ATS/ERS/JRS/ALAT in 2011 [13] by high-resolution computed tomography (HR-CT). Other inclusion criteria were: age 20 to 75 years; cytological, histological, or radiological diagnosis of non-small cell lung cancer; tolerability of general anesthesia; cancer lesion(s) resectable by single lobectomy or lesser resections; predicted post-surgical forced expiratory volume in 1 second ≥1000 mL; able to receive orally administered drugs; and Eastern Cooperative Oncology Group performance status 0–1. Patients were ineligible if they had a history of previous thoracotomy/video-assisted thoracoscopic surgery (although patients who had a history of surgical biopsy for diagnosing IPF >6 months previously were accepted); history of IPF treatment (including pirfenidone, steroids, erythromycin, N-acetyl cysteine, neutrophil elastase inhibitors, and immunosuppressants); history of radiation therapy that included a lung in the treatment field or chemotherapy; other known causes of interstitial pneumonia (e.g., environmental exposure, connective tissue disease, and drug toxicity); severe comorbidities (active infectious disease, severe heart disease, uncontrolled diabetes mellitus, gastrointestinal bleeding, glaucoma, or psychiatric disease); history of drug allergy; or obvious history of acute exacerbation of IPF. Patients receiving oxygen therapy and pregnant or breastfeeding women were also excluded.

### Study design

This was a multicenter, single-arm phase II study designed to evaluate the efficacy of perioperative pirfenidone treatment on the reduction of postoperative AE-IPF in Japanese patients with lung cancer. The primary endpoint was non–AE rate during postoperative days 0–30, and the secondary endpoint was safety. The expected non–AE rate was set at 95 %, and the non–AEthreshold was set at 80 %, because the non–AE rate in patients who did not receive perioperative pirfenidone treatment was assumed to be ≤80 % based on the literature [2–5, 14, 15]. A total of 42 patients were determined to reject the non–AE rate of 80 % under the expectation of 95 % with a power of ≥0.80 and a one-sided alpha of 0.05.

### Central review

Radiologic and pathologic diagnoses of IPF-AE were confirmed by a central review committee. To validate the institutional diagnosis of IPF, preoperative HR-CT and pathological samples obtained by surgery (non-cancerous lesions) were collected from each participating institute for central review. Two radiologists with expertise in interstitial lung disease (S.S. and T.I.) initially evaluated the HR-CT data independently and then developed a consensus report after discussion. Two pathologists with expertise in interstitial lung disease (J.F. and K.H.) initially evaluated the pathological samples independently and then developed a consensus report after discussion. These experts followed the international IPF diagnostic criteria published in 2011 [13]. The reports and discussions of the radiological/pathological diagnoses were reviewed and approved by all members of the central review committee, including the radiologists, pathologists, respiratory physicians with expertise in interstitial lung disease (A.A. and K.K.), and surgeons (S.Y. and T.I.). If the final diagnosis by HR-CT and pathological analysis was not definitively IPF, a multidisciplinary discussion was conducted among the radiologists, pathologists, respiratory physicians, and surgeons, and this group made a final diagnosis of IPF/not IPF.

Each participating institute diagnosed AE-IPF according to the definition published by the Japanese Respiratory Society [16] as follows: subjective worsening of dyspnea; new-onset bilateral ground-glass attenuation; significant worsening of partial pressure of oxygen in arterial blood; and exclusion of alternative causes of dyspnea and radiological changes, including pulmonary infection, pneumothorax, worsening of malignant tumor, pulmonary embolism, and heart failure. These criteria are very similar to the definition of the American Thoracic Society [17], although endobronchial aspiration and bronchoalveolar lavage were not required. Each incidence of postoperative AE-IPF declared by the researchers was also evaluated by the central review committee. Central review of HR-CT and clinical data were conducted through multidisciplinary discussion to determine whether or not the diagnosis of AE-IPF was appropriate.

### Drug administration

Pirfenidone (Shionogi & Co., Ltd., Osaka, Japan) at a dose of 600 mg/day was orally administered to patients who planned to undergo surgery for the first 2 weeks; the dose was then increased to 1200 mg/day for the next 2–4 weeks. The administration dose and dose-increase interval were established according to the protocol included in the Japanese pirfenidone package insert, which was based on the results of the Japanese pre-authorizing phase III trial [18]. Surgery was performed after at least 2 weeks of administration of pirfenidone 1200 mg/day. Pirfenidone was given until the morning of the surgery day, and then re-started at 1200 mg/day as soon as oral administration was permitted; in most cases, this was on the first postoperative day. The dose was increased to 1800 mg/day if possible and was continued for as long as possible at least until the 30th postoperative day.

### Analysis

In the primary analyses, the non–AE rate was based on the full-analysis set (FAS) following the intent-to-treat principle. *P*-values were calculated using binomial tests. A *p*-value <0.05 was considered significant. All statistical analysis was performed using SAS version 9.2 (SAS Institute, Cary, NC).

## Results

### Patient characteristics

From June 2012 to January 2014, 43 patients from 17 Japanese institutions were enrolled. Although all patients initiated pirfenidone therapy, 4 patients were deemed ineligible due to severe heart disease (*n* = 2), a history of chemotherapy (*n* = 1), and initiation of pirfenidone prior to registration (*n* = 1). Thus, 39 patients comprised the FAS. Patients included 33 males (84.6 %) with a median age of 68.0 (range, 52–75) years, and 38 patients had Hugh-Jones grade I/II activity (97.5 %). Among the FAS cases, two patients stopped pirfenidone administration before surgery due to patient request, and surgery was cancelled for one patient because metastatic lesions were identified on further examination. Therefore, both pirfenidone treatment and surgery were performed in 36 patients (per protocol set [PPS]). All of the PPS patients had received pirfenidone 1200 mg/day for >2 weeks at the time of surgery. The mean time at which pirfenidone was stopped around surgery was 18.4 h. Characteristics of FAS and PPS patients were very similar (Table 1). No patients had a history of gastroesophageal reflux disease. The surgical and pathological backgrounds are summarized in Table 2. Lobectomy was performed in 26 FAS patients (68.4 %) and 24 PPS patients (66.7 %). Segmentectomy was performed in seven patients (FAS, 18.4 %; PPS, 19.4 %), and wedge resection was performed in seven patients (FAS, 18.4 %; PPS, 19.4 %). Combined resections were performed in three patients (FAS, 7.9 %; PPS, 8.3 %). Histologic types included squamous cell carcinoma (FAS, *n* = 18 [47.4 %]; PPS, *n* = 18 [50 %]), adenocarcinoma (FAS, *n* = 16 [42.1 %]; PPS, *n* = 14 [38.9 %]), and other minor types. Pathological cancer stages included stage I/II (FAS, *n* = 22/12 [57.9 %/31.6 %]; PPS, 20/12 [55.6 %/33.3 %]). With respect to IPF diagnosis, the central review board diagnosed 39 of the 43 registered cases as IPF and the remaining 4 cases as not IPF (the latter were diagnosed as unclassifiable interstitial pneumonia [*n* = 2], cellular nonspecific interstitial pneumonia [*n* = 1], and respiratory bronchiolitis-associated interstitial lung disease [*n* = 1]). Compared to the central review by the professional radiologist/pathologist/respirologist, the accuracy of the preoperative radiological diagnosis of each institution was 90.7 %. Four not-IPF cases were included in the FAS and the PPS to adhere to the intent-to-treat principle. With respect to efficacy, we also selected the confirmed IPF cases from the PPS and analyzed them separately (PPS-IPF, *n* = 32).

**Table 1:** Patient characteristics

	FAS (*n* = 39)	PPS (*n* = 36)
Gender, M/F	33 (84.6 %)/6 (15.4 %)	30 (83.3 %)/6 (16.7 %)
Median age, years (range)	68.0 (52–75)	68.0 (52–74)
Smoking history, ex/never	38 (97.4 %)/1 (2.6 %)	35 (97.2 %)/1 (2.8 %)
Pack years	61.09 ± 32.07	61.85 ± 33.01
Body mass index	24.35 ± 3.43	24.41 ± 3.57
Activity		
Hugh-Jones I	29 (74.4 %)	26 (72.2 %)
Hugh-Jones II	9 (23.1 %)	9 (25.0 %)
Hugh-Jones III	1 (2.6 %)	1 (2.8 %)
Hugh-Jones IV	0 (0.0 %)	0 (0.0 %)
Hugh-Jones V	0 (0.0 %)	0 (0.0 %)
Fine crackle (audible)	17 (43.6 %)	16 (44.4 %)
Vital capacity (%predicted)	96.51 ± 15.21	97.25 ± 15.25
FEV_1.0_ (% predicted)	92.31 ± 16.74	92.81 ± 17.24
DL_CO_ (% predicted)	73.71 ± 23.11	73.88 ± 24.45
KL-6(U/mL)	1012.3 ± 891.9	1022.4 ± 924.7

*DL*_*CO*_ diffusing capacity of the lung for carbon monoxide, *FAS* full analysis set, *FEV*_*1.0*_ forced expiratory volume in 1 s, *PPS* per protocol set

**Table 2:** Patient surgical and pathological backgrounds

Surgical procedure	FAS (*n* = 38)	PPS (*n* = 36)
Operation time (hours)	3.36 ± 1.23	3.37 ± 1.25
Blood loss (g)	138.6 ± 126.2	127.7 ± 114.6
Surgical procedure		
Lobectomy	26 (68.4 %)	24 (66.7 %)
Segmentectomy	7 (18.4 %)	7 (19.4 %)
Wedge resection	7 (18.4 %)	7 (19.4 %)
Combined resection	3 (7.9 %)	3 (8.3 %)
Residual tumor		
R0	36 (94.7 %)	34 (94.4 %)
R1	1 (2.6 %)	1 (2.8 %)
R2	1 (2.6 %)	1 (2.8 %)
Cancer pathology	FAS (*n* = 38)	PPS (*n* = 36)
Pathological type		
Squamous cell carcinoma	18 (47.4 %)	18 (50.0 %)
Adenocarcinoma	16 (42.1 %)	14 (38.9 %)
Large cell carcinoma	0 (0.0 %)	0 (0.0 %)
Adenosquamous carcinoma	1 (2.6 %)	1 (2.8 %)
Other	3 (7.9 %)	3 (8.3 %)
Pathological stage		
IA	10 (26.3 %)	10 (27.8 %)
IB	12 (31.6 %)	10 (27.8 %)
IIA	8 (21.1 %)	8 (22.2 %)
IIB	4 (10.5 %)	4 (11.1 %)
IIIA	2 (5.3 %)	2 (5. %)
IIIB	0 (0.0 %)	0 (0.0 %)
IV	1 (2.6 %)	1 (2.8 %)
(non-cancer)	1 (2.6 %)	1 (2.8 %)

**Table 3:** Complications during the study

	Preoperative complications (*n* = 43)			Postoperative complications (*n* = 37)		
	Grade 1, 2	Grade 3–5		Grade 1, 2	Grade 3–5	
Photosensitivity	1	0	0.0 %	0	0	0.0 %
Anorexia	4	0	0.0 %	5	1	(Grade 4, 2.7 %)
Nausea	4	0	0.0 %	3	0	0.0 %
Diarrhea	0	0	0.0 %	1	0	0.0 %
Somnolence	1	0	0.0 %	0	0	0.0 %
Dizziness	2	0	0.0 %	1	0	0.0 %
Malaise	2	0	0.0 %	2	0	0.0 %
Respiratory failure	0	0	0.0 %	0	1	(Grade 4, 2.7 %)
Dyspnea	0	0	0.0 %	5	1	(Grade 4, 2.7 %)
Pulmonary fistula	0	0	0.0 %	2	0	0.0 %
Postoperative hemorrhage	0	0	0.0 %	1	0	0.0 %
Cough	0	0	0.0 %	1	0	0.0 %
Atrial fibrillation	0	0	0.0 %	1	0	0.0 %
Wound dehiscence	0	0	0.0 %	1	0	0.0 %
Pneumonitis	0	0	0.0 %	0	1	(Grade 5, 2.7 %)
Dysgeusia	0	0	0.0 %	1	0	0.0 %
White blood cell decreased	0	0	0.0 %	2	0	0.0 %
Neutrophil count decreased	0	0	0.0 %	1	0	0.0 %
Anemia	2	0	0.0 %	4	0	0.0 %
Hypoalbuminemia	2	0	0.0 %	19	0	0.0 %
ALP increased	2	0	0.0 %	8	0	0.0 %
Blood bilirubin increased	0	0	0.0 %	1	0	0.0 %
AST increased	3	0	0.0 %	8	1	(Grade 3, 2.7 %)
ALT increased	3	0	0.0 %	6	0	0.0 %
GGT increased	5	0	0.0 %	6	0	0.0 %
Creatinine increased	1	0	0.0 %	0	0	0.0 %
Hyponatremia	3	0	0.0 %	8	1	(Grade 3, 2.7 %)
Hyperkalemia	1	0	0.0 %	0	0	0.0 %
Hypokalemia	0	0	0.0 %	3	0	0.0 %
Hypocalcemia	0	0	0.0 %	3	0	0.0 %

*ALP* alkaline phosphatase, *AST* aspartate aminotransferase, *ALT* alanine aminotransferase, *GGT* γ-glutamyltransferase

**Table 4:** Efficacy

Group	N	AE-IPF occurred within the 30th POD	Non–AE rate within the 30th POD	95 % CI	*p*-value
FAS	39	2	94.90 %	82.7–99.4	*p* = 0.010
PPS	36	1	97.20 %	85.5–99.9	*p* = 0.004
PPS-IPF	32	1	96.90 %	83.8–99.9	*p* = 0.008

*AE-IPF* acute exacerbation of idiopathic pulmonary fibrosis, *POD* postoperative day, *FAS* full analysis set, *PPS* per protocol set, *PPS-IPF* PPS excluding non-IPF cases diagnosed by the central review committee

### Safety

The safety of pirfenidone treatment in the preoperative phase was evaluated in all patients who received pirfenidone (*n* = 43, safety analysis set) (Table 3). In the preoperative period, the following adverse events were observed: photosensitivity (*n* = 1), anorexia (*n* = 4), nausea (*n* = 4), somnolence (*n* = 1), dizziness (*n* = 2), malaise (*n* = 2), anemia (*n* = 2), hypoalubuminemia (*n* = 2), alkaline phosphatase elevation (*n* = 2), aspartate aminotransferase elevation (*n* = 3), alanine aminotransferase elevation (*n* = 3), γ-glutamyltransferase (GGT) elevation (*n* = 5), creatinine elevation (*n* = 1), hyponatremia (*n* = 3), and hyperkalemia (*n* = 1); however, all of these events were grade 1 or 2 according to the Common Terminology Criteria for Adverse Events (CTCAE) version 4.0 scale. Safety in the postoperative phase was evaluated in 37 patients in the FAS who underwent surgery. Fewer patients than those in the FAS who underwent surgery were evaluated for postoperative safety, because we did not require reporting of clinical data, except for the occurrence of AE-IPF, for patients who quit pirfenidone administration prior to surgery. The following adverse events were observed postoperatively: anorexia (*n* = 6), nausea (*n* = 3), diarrhea (*n* = 1), dizziness (*n* = 1), malaise (*n* = 2), respiratory failure (*n* = 1), dyspnea (*n* = 6), pulmonary fistula (*n* = 2), postoperative hemorrhage (*n* = 1), cough (*n* = 1), atrial fibrillation (*n* = 1), wound dehiscence (*n* = 1), pneumonitis (*n* = 1), dysgeusia (*n* = 1), white blood cell decreased (*n* = 2), neutrophil count decreased (*n* = 1), anemia (*n* = 4), hypoalbuminemia (*n* = 19), alkaline phosphatase increased (*n* = 8), blood bilirubin increased (*n* = 1), aspartate aminotransferase increased (*n* = 8), alanine aminotransferase increased (*n* = 6), GGT increased (*n* = 6), hyponatremia (*n* = 8), hypokalemia (*n* = 3), and hypocalcemia (*n* = 3). Among these, 1 case of increased aspartate aminotransferase and 1 case of hyponatremia were reported as grade 3; 1 case of anorexia, 1 case of respiratory failure, and 1 case of dyspnea were reported as grade 4; and 1 case of pneumonitis was reported as grade 5. The grade 4 respiratory failure and dyspnea and the grade 5 pneumonitis were determined to be due to AE-IPF.

Among the 43 registered patients who received pirfenidone, 41 cases underwent surgery (95.3 %). The reasons for the two surgery cancellations were discovery of metastatic lesions and necessity of cardiovascular surgery prior to lung surgery. Surgery was not cancelled in any patients due to pirfenidone-related adverse events.

### Efficacy

AE-IPF was diagnosed in two patients (94.9 % [95 % confidential interval (CI): 82.7–99.4 %, *p* = 0.01 under the null rate of 80 %]) in the FAS (Table 4). The central review board confirmed both of these cases as AE-IPF. Both patients died due to AE-IPF. One patient had quit pirfenidone administration prior to surgery; therefore, AE-IPF was recognized in only one patient (97.2 % [95 % CI: 85.5–99.9 %, *p* = 0.004 under the null rate of 80 %]) in the PPS. Among patients in the PPS, we also analyzed select IPF cases whose diagnoses were confirmed by the central review board (PPS-IPF). In the PPS-IPF, one of the 32 patients experienced AE-IPF (96.9 % [95 % CI: 83.8–99.9 %, *p* = 0.008 under the null rate of 80 %]). When compared to the threshold derived from historical controls, AE-IPF occurred at a significantly lower incidence in this study.

## Discussion

The current study was designed to evaluate the safety and efficacy of perioperative pirfenidone treatment in the reduction of postoperative AE-IPF in patients with lung cancer. No serious adverse events caused by pirfenidone were observed, and the incidence of AE-IPF among patients treated with pirfenidone was significantly lower than the null hypothesis.

The incidence of lung cancer have been reported to be >7-fold higher in IPF patients compared to the general population [1]. Conversely, IPF is detected in 3.7 % to 8 % of lung cancer patients [14, 19–21]. The prognosis of lung cancer with IPF is reported to be worse than that without IPF [14, 19, 22]. Moreover, life-threatening AE-IPF sometimes occurs as a complication of lung cancer therapy. Radiation and chemotherapy are also known to be risk factors for AE-IPF [23–25]. Although the reason for the poor prognosis of lung cancer patients with IPF is not clear, therapeutic limitations associated with the potential of AE-IPF might at least partially account for the higher recurrence rate. If an effective strategy to reduce AE-IPF is established, more rational and radical treatment could be delivered to patients in a safer manner, which would lead to improved patient outcomes.

No studies have yet demonstrated that any agents can prevent or decrease the incidence of postoperative AE-IPF [20]. Pirfenidone is the first anti-fibrotic agent approved for the treatment of IPF [18]. The pooled data from the ASCEND and CAPACITY [26] trials showed that death related to IPF was significantly reduced among patients receiving pirfenidone therapy [12]. A phase II Japanese study found that pirfenidone prevented the development of AE-IPF [27]. However, these randomized studies evaluated the effect of pirfenidone in the context of the natural history of IPF. The effects of pirfenidone in patients undergoing anticancer therapy such as surgery, radiation, and chemotherapy, which are all risk factors for AE-IPF, have previously been unknown. We evaluated perioperative pirfenidone treatment within a single institute, and the results of this experience with a small sample size suggested that it is a feasible treatment and can be expected to reduce the incidence AE-IPF [28]. Therefore, we planned this multi-institutional prospective study to confirm our previous results.

Although AE-IPF is a well-known, life-threatening postoperative complication in patients with lung cancer accompanied by IPF, its reported incidence varies among studies; however, it generally ranges from 10 % to 20 % [2–5, 7, 14, 15, 19, 29, 30]. Compared to many small studies that evaluated 10–60 cases, Yoshimura et al. [5] reported the largest series of 205 IPF patients with lung cancer. In this study, these investigators observed that 41 patients (20 %) developed AE-IPF postoperatively, among which 29 (71 %) died of respiratory failure due to AE-IPF. In the current study, an 80 % non-exacerbation rate was set as the null hypothesis based on these previous reports, which were published prior to the planning of the current protocol in 2010; the non–AE rates in the current study of 94.9 % (FAS), 97.2 % (PPS), and 96.9 % (PPS-IPF) were statistically positive compared to this null hypothesis.

Several studies have revealed that interstitial pneumonia subtype (UIP pattern or not) is a risk factor for postoperative AE-IPF [10, 31]. However, interstitial pneumonia subtypes were diagnosed with varying diagnostic criteria among different institutions in these previous studies. To circumvent this problem, we introduced a central review system to confirm the diagnosis of IPF following the international guidelines. As a result, we were able to show ~90 % diagnostic accuracy for each of the participating institutes. We believe that this first multi-institutional prospective study to evaluate postoperative AE-IPF after lung cancer surgery revealed reliable objective data about the incidence of AE-IPF; these data can be used as a standard when designing future studies.

### Limitations

This study was not randomized but rather was a single-arm study in which historical data served as the control. When we combined the Japanese and international data for surgical lung cancer patients with idiopathic interstitial pneumonia published prior to the planning of the present study, the total incidence of AE was about 19 % (data not shown); therefore, we believe the null hypothesis set herein was appropriate. However, patient background and the influence of some types of patient management that may have been better than those reported in the literature could not be controlled in such a single-arm study. Because the reported background risks for AE also varied among studies, it is difficult to verify the difference between the background risk in this study and those of previous studies. For example, respiratory function and activity shown by Hugh-Jones classification of the registered patients were relatively good in the current study. Severe IPF is undoubtedly considered to be a high risk; however, functionally inoperable cases were not included in this surgical study, and in many cases IPF was newly detected by surgeons during preoperative examination. Perhaps due to such a situation bias, the previously reported preoperative respiratory functions were also almost normal [4, 14, 15, 19, 28–31]. Postoperative AE occurs despite such surgical selection bias. To overcome such background problems and to confirm the efficacy of perioperative pirfenidone treatment for postoperative AE-IPF, a randomized study is required. Despite this limitation, we believe that the positive results of this phase II study justify the planning of a phase III randomized study.

## Conclusions

In conclusion, this single-arm phase II study revealed that perioperative pirfenidone treatment is safe and promising for reducing AE-IPF after lung cancer surgery. These results encourage the planning of future confirmatory studies to compare pirfenidone to other treatments, such as nintedanib, for which the efficacy against progression of IPF has already been reported [32].

## Abbreviations

AE: acute exacerbation; FAS: full analysis set; HR-CT: high-resolution computed tomography; IPF: idiopathic pulmonary fibrosis; JACS: Japanese Association of Chest Surgeons; PPS: per protocol set; UIP: usual interstitial pneumonia; WJOG: West Japan Oncology Group
